# Editorial: Insights, controversies, and new developments in the initial treatment decisions for advanced epithelial ovarian cancer

**DOI:** 10.3389/fonc.2025.1568579

**Published:** 2025-03-03

**Authors:** Jianfeng Guo

**Affiliations:** ^1^ Department of Obstetrics and Gynecology, People’s Hospital of Longhua, Shenzhen, China; ^2^ Longhua District Key Laboratory of Perinatal Population Medicine, Shenzhen, China

**Keywords:** epithelial ovarian cancer (EOC), initial treatment decisions, machine learning in oncology, homologous recombination deficiency (HRD), patient-derived organoids (PDOs), platinum resistance, PARP inhibitors (PARPi), three-dimensional ultrasonography

## 1 Research background and significance

Epithelial Ovarian Cancer (EOC) is one of the deadliest types of gynecological malignancies, especially in advanced cases, and the five-year survival rate remains low. Despite recent advances in surgical techniques, chemotherapy regimens, and targeted therapies, initial treatment decision making remains challenging. How to balance the effect of treatment and the quality of life of patients, how to find the best balance between individualized treatment and standardized treatment, is the core problem in current clinical practice.

## 2 Feature article overview

This topic includes a number of high-quality research articles, covering a wide range of aspects from basic research to clinical practice. Here is a brief overview of a few representative articles:

### 2.1 *Machine learning for epithelial ovarian cancer platinum resistance recurrence identification using routine clinical data* (Yang et al.)

This study developed machine learning models to predict platinum-resistant recurrence in epithelial ovarian cancer (EOC) using clinical and laboratory data from 1,392 patients. Five algorithms (DTA, KNN, SVM, RF, XGBoost) were compared, with XGBoost based on multiple logistic regression showing the best performance (AUC: 0.784, accuracy: 80.4%). Key variables influencing recurrence were identified, and models were visualized via nomograms for clinical use. The findings highlight the potential of machine learning in improving EOC recurrence prediction, though continuous model updates are needed to adapt to evolving clinical contexts.

### 2.2 *Incidental findings of borderline ovarian tumor or ovarian cancer – real-world data on surgical and oncological outcomes* (Joder et al.)

This study compared surgical and oncological outcomes in patients with incidental borderline ovarian tumors or ovarian cancer treated with a two-stage surgical procedure versus those with suspected malignancy undergoing a single-stage procedure. Among 223 patients, those with incidental diagnoses had more surgeries and longer intervals to chemotherapy and cytoreduction completion. However, there were no significant differences in complete cytoreduction rates, complication rates, hospitalization days, recurrence risk, or survival. Despite delays, incidental diagnosis and two-stage treatment did not negatively impact oncological outcomes, supporting their feasibility in clinical practice.

### 2.3 *Pathological PCI as a prognostic marker of survival after neoadjuvant chemotherapy in patients undergoing interval cytoreduction with or without HIPEC in FIGO stage IIIC high grade serous ovarian cancer* (Sinukumar et al.)

This study evaluated pathological PCI (pPCI) as a prognostic marker for survival in high-grade serous ovarian cancer patients treated with neoadjuvant chemotherapy and interval cytoreductive surgery. Among 171 patients, ROC analysis identified a pPCI cut-off of 8 as optimal for predicting survival (sensitivity: 82%, specificity: 67%). Patients with pPCI ≥8 had worse overall and disease-free survival (p=0.002 and p=0.001, respectively). Despite complete cytoreduction in 88% of cases, pPCI ≥8 emerged as a poor prognostic indicator, suggesting its utility in guiding adjuvant treatment decisions, particularly in resource-limited settings.

### 2.4 *Advances and challenges in the origin and evolution of ovarian cancer organoids* (Zhang et al.)

Epithelial ovarian cancer, with a five-year survival rate of 48%, remains a significant threat, especially for advanced FIGO III-IV cases. Despite standard treatments, recurrence and chemoresistance are common. Patient-derived organoids (PDOs), a 3D cancer model, have revolutionized research by replicating disease characteristics and enabling personalized treatment strategies. PDOs predict treatment responses, facilitate drug testing, and uncover drug resistance mechanisms, offering insights into cancer progression. This review highlights PDOs as a transformative tool in ovarian cancer research, bridging foundational theories and clinical applications to advance therapeutic development and individualized care.

### 2.5 *Limitations of homologous recombination status testing and poly (ADP-ribose) polymerase inhibitor treatment in the current management of ovarian cancer* (Zhao et al.)

Homologous recombination deficiency (HRD), marked by mutations in BRCA1/2 and genomic scars, is a stable biomarker in ovarian cancer (OC). PARP inhibitors (PARPi) exploit HRD via synthetic lethality, showing significant benefits but limited by resistance and unclear long-term survival impact. Current HRD scoring lacks comprehensiveness, especially in HR-proficient cases. Beyond BRCA, understanding HR gene interactions and alternative pathways is crucial for refining PARPi efficacy and developing precise strategies for advanced, recurrent, and refractory OC patients. Further research is needed to optimize HRD’s role in treatment and resistance management.

### 2.6 *Is three-dimensional ultrasonography a valuable diagnostic tool for patients with ovarian cancer? Systematic review and meta-analysis* (Liu et al.)

This study evaluated the diagnostic performance of 3D ultrasonography (3DUS), 3D power Doppler (3DPD), and their combination in ovarian cancer (OC). Analyzing 18 studies (2,548 cases), 3DUS showed sensitivity of 0.89 and specificity of 0.93, while 3DPD had sensitivity of 0.90 and specificity of 0.85. Combining 3DUS and 3DPD achieved superior diagnostic efficiency, with sensitivity of 0.99, specificity of 0.95, and AUC of 0.99. The findings suggest that 3DUS and 3DPD, particularly when combined, are highly effective diagnostic tools for OC, offering improved accuracy and clinical utility.

## 3 Controversy and future direction

Although the articles on this topic have provided us with a wealth of insights, there are still many disputes that need to be resolved. For example, how can we better integrate targeted therapy with immunotherapy in the initial treatment? How to further optimize individual treatment strategies through multi-omics analysis? In addition, with the accumulation of real-world data, how to translate these data into decision support tools in clinical practice is also an important direction for future research.

## 4 Conclusion

Overall, this topic provides new ideas and evidence for the initial treatment decision of advanced epithelial ovarian cancer through multi-angle discussion. We expect these findings to provide additional references for clinicians and ultimately improve patient outcomes and quality of life.” In the future, with more high-quality research, we believe that the treatment of advanced EOC will have a brighter future.

